# Erratum for “Use of Indices Combining Diastolic and Systolic Tissue Doppler Variables to Evaluate Right Ventricular Function in Dogs With Pulmonary Stenosis”

**DOI:** 10.1111/jvim.70065

**Published:** 2025-03-20

Winter, R.L., Maneval, K.L. and Ferrel, C.S. (2025), Use of Indices Combining Diastolic and Systolic Tissue Doppler Variables to Evaluate Right Ventricular Function in Dogs With Pulmonary Stenosis. J Vet Intern Med, 39: e70022. https://doi.org/10.1111/jvim.70022.

In Table 3, the first row of data for “RV E (cm/s)” in first, second and third columns need correction.

First column “Healthy (n = 15)” should display “65 (48‐110) ^a^”

Second column “PS pre‐op (n = 15)” should display “49 (38‐81) ^a^”

Third column “PS post‐op (n = 15)” should display “58 (38–90)”

This does not change anything in the paper, including results from statistical analysis. The correct numbers were used for analysis.

